# Supplementary material for: Antihypertensive Effects of Gynura divaricata (L.) DC in Rats with Renovascular Hypertension
Source: Nutrients. 2020 Oct 29;12(11):3321. doi: 10.3390/nu12113321 (PMC7692656; doi:10.3390/nu12113321)
Supplement: Supplementary file 1 [file nutrients-12-03321-s001.pdf]

Supplementary Materials.

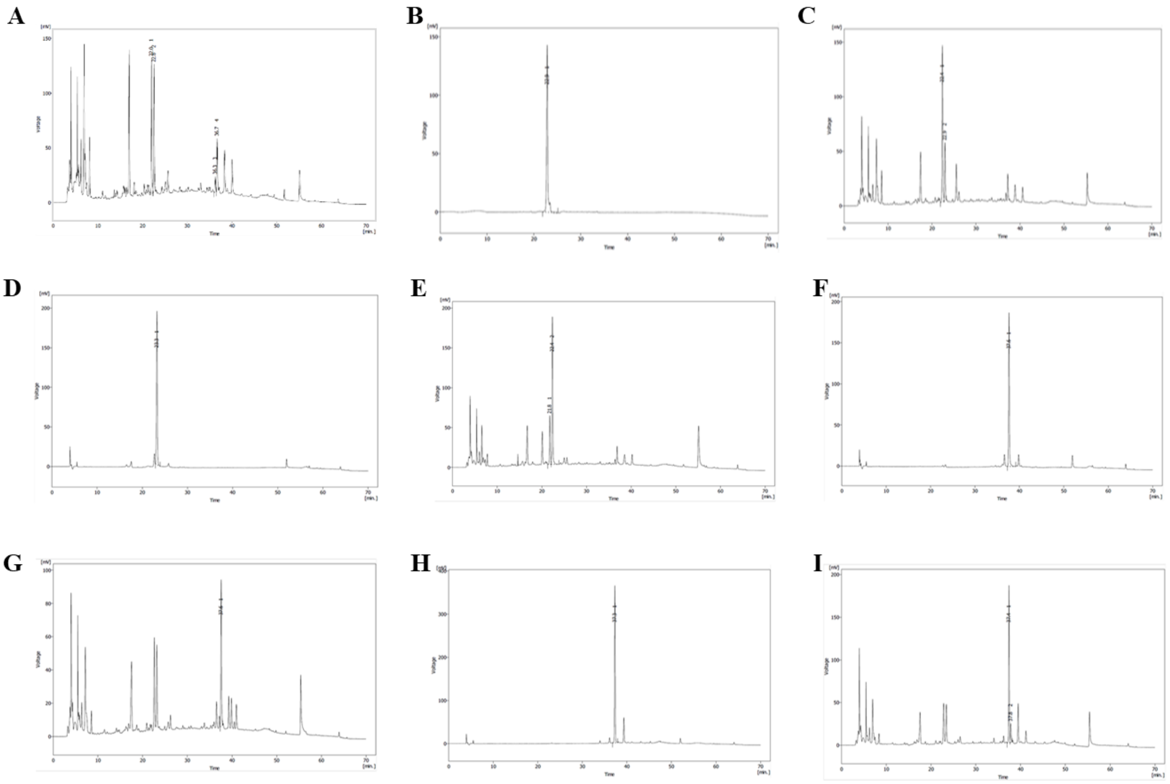

Supplementary Figure 1: High-performance liquid chromatography (HPLC) chromatograms of ***Gynura divaricata*** **(L.)** DC (GD). (**A**) GDa; (**B**) GDB2-3; (**C**) GDB2-3 with GDa; (**D**) GDB2-4; (**E**) GDB2-4 with GDa; (**F**) GDB4-1; (**G**) GDB4-1 with GDa; (**H**) GDB4-4; (**I**) GDB4-4 with GDa,.

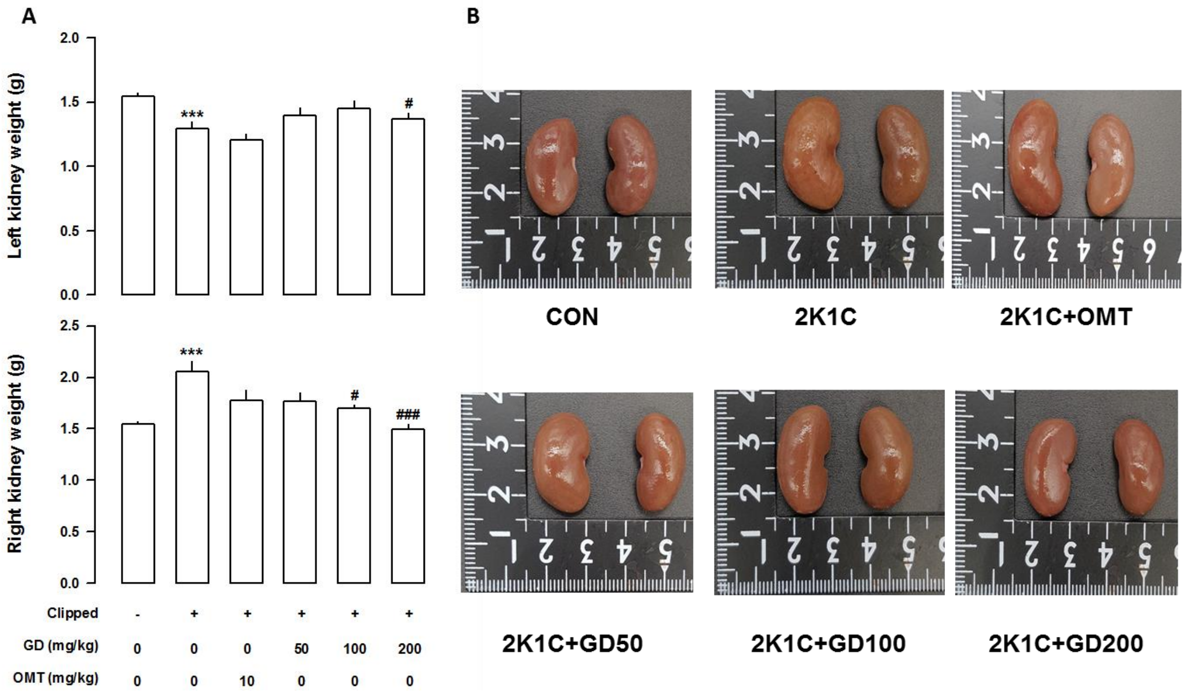

Supplementary Figure 2: Effects of *Gynura divaricata* (L.) DC (GD) on kidney weight and shape. (**A**) Effect of GD on the left and right kidney weight; (**B**) Kidney size. Each value shows the mean ± standard error (n = 10 per group). *** $p < 0.001$, vs. CONT and $^{\#}$ $p < 0.05$, $^{\#\#\#}$ $p < 0.0001$ vs. 2K1C.

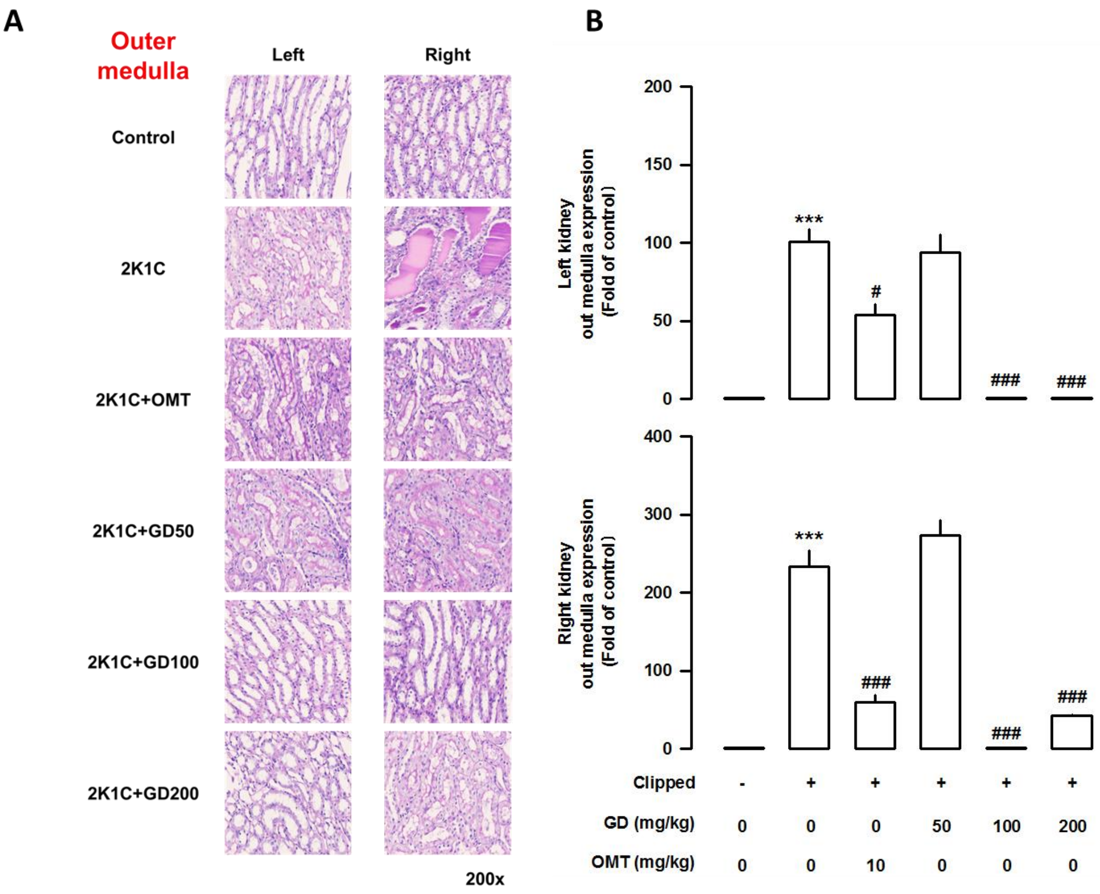

Supplementary Figure 3: Effects of *Gynura divaricata* (L.) DC (GD) on kidney outer medullar fibrosis. (**A**) Periodic acid-Schiff staining of the kidney in the outer medulla (magnification × 200); (**B**) Quantitative assessments, representing the results of five independent experiments. The values are expressed as the mean ± standard error (n = 5 per group). *** $p < 0.001$, vs. CONT and $^{\#}$ $p < 0.05$, $^{\#\#\#}$ $p < 0.0001$ vs. 2K1C.

Supplementary Table 1. EC_{50} and E_{max} values for ACh, SNP, and ANP-induced relaxation of GD treatment in 2K1C rats.

	ACh		SNP		ANP	
	E_{max} (%)	log EC_{50} (M)	E_{max} (%)	log EC_{50} (M)	E_{max} (%)	log EC_{50} (M)
**cont**	93.70 ±2.83	-8.81 ±0.05	91.34 ±0.54	-8.99 ±0.03	80.60 ±2.00	-8.44 ±0.28
**2K1C**	59.61 ±2.22***	-7.79 ±0.09*	77.72 ±3.77**	-8.14 ±0.07*	54.64 ±7.28**	-8.00 ±0.31*
**2K1C+OMT**	92.22 ±3.79$^{\#\#}$	-8.45 ±0.17$^{\#}$	97.72 ±1.52$^{\#\#\#}$	-8.88 ±0.02$^{\#}$	88.91 ±2.71$^{\#\#\#}$	-8.69 ±0.06$^{\#}$
**2K1C+GD50**	76.45 ±3.46	-8.09 ±0.07	84.71 ±2.76	-8.66 ±0.05	72.85 ±4.49$^{\#}$	-8.51 ±0.08$^{\#}$
**2K1C+GD100**	86.28 ±3.12$^{\#\#}$	-8.12 ±0.08	90.96 ±2.88$^{\#}$	-8.84 ±0.03$^{\#}$	80.09 ±4.17$^{\#\#}$	-9.36 ±0.10$^{\#}$
**2K1C+GD200**	92.72 ±4.08$^{\#\#}$	-9.03 ±0.14$^{\#}$	88.74 ±2.43$^{\#}$	-8.94 ±0.03$^{\#}$	76.41 ±4.07$^{\#}$	-9.36 ±0.09$^{\#}$

EC50 and Emax values of ACh, SNP, and ANP were calculated of the relaxation-response curves to GD treatment in 2K1C rats. Values are means ±SE of 6 experiments for EC_{50} and E_{max}. *$p < 0.01$, **$p < 0.01$ ***$p < 0.01$ vs. Cont.; # $p < 0.05$, ##$p < 0.01$, and ### $p < 0.001$ vs. 2K1C.
